# Inter-class Concomitant Pharmacotherapy in Medicaid-Insured Youth Receiving Psychiatric Residential Treatment

**DOI:** 10.3389/fpsyt.2021.658283

**Published:** 2021-05-20

**Authors:** Gail A. Edelsohn, Kemal Eren, Meghna Parthasarathy, Neal D. Ryan, Amy Herschell

**Affiliations:** ^1^Community Care Behavioral Health Organization, University of Pittsburgh Medical Center (UPMC) Insurance Services Division, Pittsburgh, PA, United States; ^2^Department of Psychiatry, University of Pittsburgh, Pittsburgh, PA, United States

**Keywords:** concomitant pharmacotherapy, polypharmacy, children and adolescents, Medicaid, residential treatment

## Abstract

**Background:** Concomitant pharmacotherapy has become increasingly common in the treatment of youth, including in psychiatric residential treatment facilities (PRTF) despite limited efficacy and safety data. Research is reported on the prevalence of any class and interclass concomitant pharmacotherapy, specific class combinations of psychotropics, and changes in number of medications from admission to discharge for Medicaid insured youth treated in PRTFs in one mid—Atlantic state.

**Methods:** Medicaid administrative claims data were examined for youth under age 18 years who were discharged from one of 21 PRTFs during calendar year 2019. Descriptive statistics were calculated to examine patterns of service utilization 90 days prior to admission. The rates of concomitant psychotropic use at admission were compared to the rates at discharge. Logistic regression models were used to examine covariates associated with discharging on 4 or more medications.

**Results:** Fifty-four % of youth were admitted on either two or three psychotropics, while 25% were admitted on four or more psychotropics. The proportion of youth admitting and discharging on 2 or 3 medications was stable. There was a 27% increase in number of youth discharging on 4 medications with a 24% decrease in those on a 5- drug regimen. Only the number of medications prescribed at admission was found to be significant (*p* < 0.001), with more medications at admission contributing to probability of discharging on 4 or more medications.

**Conclusions:** Concomitant pharmacotherapy is common in PRTFs. These findings support the practice of deprescribing and underscore the need for further research.

## Background

Concomitant psychotropic use, also referred to as polypharmacy, has become widespread and data suggest that it is increasing (1–4). A 29 state Medicaid fee-for-service study found the prevalence of any class and inter-class polypharmacy increased from 21.2 and 18.8% in 1999–2000 to 27.3 and 24.4% in 2009–2010, respectively in medicated youth <18 years of age (4). A single state retrospective cohort study using 2012–2015 claims data found 38% of youth on psychotropic medication for at least 90 days were prescribed two or more psychotropic medications during the study period (1). A cross sectional study used national household survey data, and found the number of youths younger than 18 years treated with three or more psychotropic classes increased from 101,836 (1999–2004) to 293,492 (2011–2015) (5).

Concomitant psychotropic pharmacotherapy has been utilized as a strategy to address residual symptoms and incomplete response to monotherapy, to augment response to other psychotropics, to mitigate side effects of concurrent medication, to treat medical comorbidity and to increase medication tolerability (6). The combination of multiple medications may offer benefits for specific clinical conditions such as the use of a stimulant and alpha agonist for the treatment of attention deficit hyperactivity disorder (ADHD); however, overall the evidence base for concomitant pharmacotherapy in pediatric psychiatric practice is limited while risk for harm is significant (7). Using concomitant psychotropics increases the risk of for drug-drug interactions, additive drug adverse reactions, and may create a cycle of using one drug to treat the adverse effects of another. Another concern is the unknown impact of long-term exposure (7–10). Burcu et al. (10) found twice the of risk of type 2 diabetes among Medicaid youth receiving concurrent atypical antipsychotics with SSRI/SNRI antidepressants. A series of case reports of presumed serotonin syndrome in children receiving multiple concurrent psychotropics including SSRIs, stimulants, and atypical antipsychotics underscores the risk of serotonin toxicity when prescribing multiple medications (11–13). There is emerging evidence that many classes of psychotropic medications can have a negative impact on pediatric bone health and increase the risk for osteoporosis (14). The most evidence exists for anticonvulsant mood stabilizers prompting recommendations for routine baseline and monitoring of vitamin D levels for all children on anticonvulsants and vitamin D supplementations for those on chronic mood stabilizers (15). As atypical antipsychotics and SSRIs can increase prolactin levels through different mechanisms, it is important to monitor bone health for children prescribed combinations of antipsychotics, mood stabilizers and SSRIs (14).

Psychiatric residential treatment facilities (PRTFs) are non-hospital facilities that provide intensive, 24/7 level treatment under the direction of physician with a range of therapeutic interventions including psychopharmacotherapy provided by a multidisciplinary team. PRTF services are an optional Medicaid benefit serving individuals up to age 21years. Youth entering a PRTFs share a history of multiple episodes of treatment, including community-based services and acute inpatient mental health hospitalization, and concomitant psychotropic use.

Studies of concomitant psychotropic use in residential settings are heterogeneous with regards to types of facilities such as child welfare and/or juvenile justice placements, group homes, residential treatment centers and PRTFs. Residential settings vary with regards to census, population characteristics, intensity, types of treatment offered, and dedicated psychiatric time. Connor et al. (16) found that 76% of youth admitted to a residential treatment center were on psychotropics, with 40% on more than one psychotropic medication. A retrospective naturalistic study of a single residential treatment setting over a 9-year period found that the number of children on multiple concurrent medications decreased from 78% at admission to 48% at discharge (17). Factors correlated with a reduction in medication included a decrease in psychopathology scores, youth admitted from more intact families (biological or adoptive), and those treated with non-stimulant medications. A retrospective study of 1,010 youth at a large group-home facility during 2001–2004 found a decrease from 40% on any medications at admission to 26% on no medications at discharge. Several studies published from 2013 to 2016 reported a reduction of psychotropics and concomitant pharmacotherapy with the use of structured psychosocial treatment programs and implementation of evidence-informed prescribing practices (18–21). Bellonci et al. (18) study of two different residential treatment centers found a decrease in the average numbers of psychotropics from admission (3.5) to discharge (1.4) with improved outcomes. Of note the psychiatrists at both sites embraced the principle of sufficiency with regards to medication use. Lee et al. (20) 10-year study (2003–2012) found a 26% cost reduction in psychotropic medications at a juvenile justice residential treatment program implementing psychiatric practice guidelines without an increase in aggression compared to cost increases found (104%, 152%) at two comparison programs.

Community Care Behavioral Health Organization (CCBHO), part of the UPMC Insurance Services Division, is a non-profit behavioral health managed care organization (BHMCO) that manages behavioral health services for 41 of Pennsylvania's 67 counties, which represents 38% of all Medicaid members in Pennsylvania. Given the expanded use of concomitant pharmacotherapy in youth, and the limited evidence for efficacy and safety, the BHMCO examined utilization of concomitant psychotropic medications in the Medicaid- enrolled pediatric population receiving services in PRTFs. First, we describe the utilization of concurrent psychotropic medication in PRTFs by gender, race, ethnicity, age group, and diagnosis. Second we compare changes in both number of medications and the number of concurrent classes of medications from admission to discharge. We report the prevalence of inter-class concomitant pharmacotherapy for 2, 3, and 4 or more medication classes and the common combinations of classes of medications. Third, we test a model to examine if covariates are associated with being discharged on four or more medications.

## Method

### Data Source

Medicaid administrative claims data were examined for 548 Medicaid enrolled youth <18 years within the BHMCO network who discharged from a PRTF in calendar year 2019. Claims from all 21 PRTFs across the BHMCO's network with eligible discharges were included. The study was approved through UPMC Quality Review Committee.

### Study Measures

#### Sociodemographic Characteristics

Demographic information including age, gender, race, and ethnicity were derived from administrative eligibility data from the state. Youth were categorized by age group into 6–12 and 13–17 years. Race and ethnicity were recorded from self-reported eligibility information. Race was categorized as Black, White or other; ethnicity was recorded as Hispanic/non-Hispanic.

#### Diagnosis Groupings and Behavioral Health Services

Diagnoses (ICD 10 codes) were obtained from claims data. Up to three admitting diagnoses were recorded from each claim. Diagnoses were categorized into the following groups: Disruptive Mood Dysregulation Disorder (DMDD), Attention Deficit Hyperactivity Disorder (ADHD), Mood Disorders (major depressive disorders and persistent mood disorders), Anxiety, Trauma and Stress Disorders, Disruptive, Impulse and Conduct Disorders, Autism Spectrum Disorder (ASD), and Psychotic Disorders. A list of ICD-10 codes is provided in Supplemental Table 1. As DMDD is a relatively recent diagnosis without an FDA- indicated treatment medication, it was decided to not incorporate it into another diagnostic grouping. The diagnostic groups are not mutually exclusive, and each youth can potentially be counted in more than one diagnostic group. All behavioral health service claims data in the 90 days prior to admission to PRTF were grouped into broad categories: outpatient services, school and community- based programs, case management, medication management, substance use treatment, partial hospital programs and inpatient mental health.

Receipt of a behavioral health service was considered as any claim for that service in the 90 days prior to PRTF admission. We dichotomized youth into those who had IPMH treatment over that interval and those who did not. A youth could be counted in more than one service level as he/she could have had multiple claims and multiple services in the 90 days prior to PRTF admission, but the youth is only assigned to one group (with or without IPMH).

#### Medications

Psychotropic medication data were obtained from paid pharmacy claims. All psychotropics were categorized into the following classes: antipsychotics, antidepressants, alpha- 2-agonists, mood stabilizers, stimulants, melatonin, anticholinergic agents, antianxiety medication, atomoxetine, benzodiazepines, substance use disorders medications, antihistamines and hypnotics. Lithium was included in the class of mood stabilizers. Diphenhydramine was the only antihistamine identified. A list of medications within subgroups is provided in Supplemental Table 2.

#### Definition of Concomitant Psychotropic Use

Concomitant psychotropic use was defined as the presence of at least 2 concurrent psychotropic medication prescription dispensing events. Inter-class pharmacotherapy was defined as at least 2 different classes of medication that would be used at the same time for at least 60 consecutive days. The allowable gap for medication fills was 7 days. Paid pharmacy claims at 30 days post admission and at 30 days prior to discharge served as proxies for admission and discharge medications. The requirement of at least 60 consecutive days to qualify as concurrent psychotropic use was applied for the entire duration with the exception of the first and last 30 days.

#### Covariates

We obtained the following covariates on all participants: age at admission, race, ethnicity, gender, length of stay in days, inpatient mental health services (IPMH) in the 90 days prior to residential treatment admission, number of medications at admission, number of medications at discharge, diagnoses, and Medicaid eligibility groups.

#### Statistical Analysis

Descriptive statistics were calculated to examine demographic data (age groups, race, gender), length of stay, and history of behavioral health services in the 90 days prior to admission to the PRTF. The most common combinations of medication classes were determined for inter-class concurrent medications. Prevalence rates for the number of youth dispensed any psychotropics as well as the number of youth dispensed inter-class combinations were obtained for admission and discharge medications.

L1 regularized regression (LASSO regression) is a linear regression model with an additional penalty term to encourage sparsity of the coefficients (22). It can therefore be used as a type of features selection because it drops unnecessary covariates, such as those that are highly correlated, those with a small effect size, or those that are not predictive of the outcome. Logistic LASSO regression models were used to predict discharging on 4 or more medications. Data were restricted to the youth that fit the criteria. The regularization parameter was set to alpha =5. The following covariates were included in the model: number of medications at the time of admission, length of stay, inpatient mental health treatment 90 days prior to admission, race, age >13 years, Medicaid eligibility groups, and the presence of any of the following diagnoses: DMDD, bipolar disorder, ASD, and psychotic disorders.

## Results

### Demographic and Clinical Characteristics of Youth at Admission to PRTF

Table 1 presents the demographic and clinical characteristics of youth as well as the behavioral health services received in the 90 days prior to admission to PRTF. Adolescents accounted for 70% of youth admitted. All youth received a range of behavioral health services prior to admission, and 50% were treated in an inpatient mental health service in the previous 3 months.

**Table 1:** Demographic and clinical characteristics of youth at time of admission to PRTF.

**Categories**	**Total**	**Percent**
	***N* = 548**	
**Age groups**		
6–12	166	30.3%
13–17	382	69.7%
**Race**		
Black	97	17.7%
White	410	74.8%
Other	41	7.5%
**Ethnicity**		
Non-Hispanic	512	93.4%
Hispanic	36	6.6%
**Gender**		
Female	248	45.3%
Male	300	54.7%
**Diagnostic groups**		
Disruptive mood dysregulation disorder	121	22.1%
Attention-deficit hyperactivity disorder	120	21.9%
Mood disorders (major depressive disorder, persistent depressive disorder)	116	21.2%
Anxiety, trauma, stress disorders	113	20.6%
Disruptive, impulse & conduct disorders	98	17.9%
Other	81	14.8%
Bipolar disorder	74	13.5%
Autism spectrum disorder	54	9.9%
Psychotic disorders	10	1.8%
**Medicaid eligibility groups**		
SSI	255	46.5%
TANF	191	34.9%
THM	102	18.6%
**BH services 90 prior to admission**		
Inpatient mental health (IPMH)+ other services	274	50%
BH services without IPMH	274	50%

*SSI, supplemental security income; TANF, temporary assistance for needy families; THM, TANIF, healthy horizons, and MAGI (modified adjusted gross income); BH, behavioral health*.

### Number of Medications and Classes of Medications at Admission and Discharge

Table 2 presents the rates of specific medication classes prescribed at time of admission with antipsychotics (62%) antidepressants (53%), and alpha−2-agonists (37%) being the most prevalent.

**Table 2:** Psychotropic medications at admission.

**Medication class**	**Number of children**	**% (Den = 548)**
Atypical antipsychotics	341	62.20%
Antidepressants	290	52.90%
Alpha-2 agonist	202	36.90%
Mood stabilizers	190	34.70%
Stimulants	136	24.80%
Melatonin	50	9.10%
Antihistamines	27	4.90%
Anticholinergics	24	4.40%
Anti-anxiety meds	23	4.20%
Atomoxetine	23	4.20%
Benzodiazepines	19	3.50%
SUD/alcohol meds	5	0.90%
Hypnotics	4	0.70%

Changes in the number of medications prescribed at admission compared to the number of medications prescribed at discharge are shown in Table 3. Of the 548 youth who were discharged from PRTFs in CY 2019, 54.3% were admitted on either 2 or 3 psychotropic medications, while 25% of youth were admitted on 4 or more medications. The number of youth admitting and discharging on one medication decreased slightly. There was little change in the number of youth on 2 or 3 medications from admission to discharge. There was a 27% increase in number of youth discharging on 4 medications, with a 24% decrease in the number of youth discharging on a 5 or more-drug regimen. The pattern of changes from admission to discharge for number of inter-class combinations was stable for 2 and 3 inter-class combinations, with a 6% increase in number of youth discharging on 4 or more inter-class combinations.

**Table 3:** Changes in number of medications from admission to discharge.

	**Number of medications**					
	**0**	**1**	**2**	**3**	**4**	**5 or more**
Number of youth at admission	41	72	143	155	83	54
Number of youth at discharge	36	69	145	152	105	41

### Duration of Medications, Inter-class Combinations and Same Class Concurrent Psychotropics

Of the 548 youth, 86.9% received 2 or more different psychotropics for at least 60 days, with a median duration of 110 days (range of 60–1,114 days). 64.6% of youth received 3 or more different psychotropics for at least 60 days with a median of 109 days (range 60–917 days). For children receiving 3 or more different classes of medication, 55.7% received these medications for 90 days or more with a median of 146 days (range 90–917 days).

Table 4 shows the most common combinations of classes of medications found for each level of inter-class concomitant psychotropic use. Antidepressants, alpha-agonists, and antipsychotics were the most frequently combined classes present in three concurrent inter-class psychotropics, followed by mood stabilizers and stimulants. Data for combinations prescribed to fewer than 5 children were grouped as “Other” revealed many different combinations too numerous to list. Twenty-two percent of youth received same class concurrent medication with antidepressants being most frequent (13%), antipsychotics and mood stabilizers (3%) each, respectively, while alpha-2-agonists, stimulants, and anticholinergic medications were negligible.

**Table 4:** Most common drug classes in concurrent medications prescribed at discharge.

**Concurrent medication**	**Most common**	**Total children**	**% of group**
	**drug classes**	**(*N* = 548)**	
2 medication	(AD, AP)	37	22.42
classes (*N* = 165)	(AA, AP)	20	12.12
	(AP, MS)	17	10.30
	(AA, AD)	16	9.70
	(AD, MEL)	9	5.45
	(AP, STM)	9	5.45
	(AD, MS)	8	4.85
	(AD, STM)	8	4.85
	(AA, MS)	5	3.03
	Other	36	21.82
3 medication	(AA, AD, AP)	18	10.98
classes (*N* = 164)	(AD, AP, MS)	17	10.37
	(AA, AP, MS)	15	9.15
	(AA, AP, STM)	12	7.32
	(AA, AD, STM)	11	6.71
	(AD, AP, STIM)	10	6.10
	(AD, MS, STM)	6	3.66
	(AA, AD, MEL)	6	3.66
	(AA, AD, MS)	5	3.05
	Other	64	39.02
>4 medication	(AA, AD, AP, STM)	10	9.52
classes (*N* = 105)	(AA, AD, AP, MS)	8	7.62
	(AA, AD, MS, STM)	6	5.71
	Other	81	77.14

*AD, antidepressant; AP, antipsychotic; AA, alpha agonist; MS, mood stabilizer; MEL, melatonin; STM, stimulant; Other, combinations of medication classes prescribed to fewer than 5 children*.

### Covariates Associated With Discharge on 4 or More Medications

The logistic regression model to predict discharging on 4 or more medications included the following variables: age in years, race, ethnicity, indicator for any inpatient mental health service 90 days prior, number of medications on admission, length of stay (days), Medicaid eligibility groups, and admitting or discharging with a diagnosis of DMDD, or bipolar disorder or ASD, or psychotic disorder.

Only the number of medications prescribed at admission was found to be significant (*p* < 0.001), with more medications at admission contributing to probability of discharging on 4 or more medications.

## Discussion

Our finding that 54% of youth were admitted on either 2 or 3 psychotropic medications, while 25% of youth were admitted on 4 or more medications aligns with previous reports of the prevalence of concomitant pharmacotherapy prior to or at the time of admission to residential settings (16, 18–21, 23). Children served in the public sector, in foster care, those who have experienced trauma, and those with intellectual disability are vulnerable to high rates of concomitant psychotropic medication (7, 24–26).

PRTFs offer a longer duration of multimodal treatment in a structured therapeutic setting conducive to re-evaluating prior interventions and obtaining multiple observation points in time to assess the benefits and risks of medications. However, we did not find a reduction in concomitant prescribing over the course of treatment in PRTFs. Most youth were discharged on the same number of medications on which they entered. Medications tended to be added if a youth was admitted on three or less medications and reduced if a youth was admitted on five or more medications. It is quite possible the dosage of medications would have been titrated over the course of treatment or that specific medications would have changed during the treatment episode; however, we did not obtain that information for this report. Clinical practice guidelines typically target the treatment of single disorders, leaving prescribing clinicians without adequate tools to address the increased use of concomitant pharmacotherapy. Youth in our sample received multiple psychiatric diagnoses, a finding congruent with previous reports of the complex behavioral health needs of youth in residential treatment settings. Bellonci et al. (18) highlight several challenges facing psychiatrists working in residential treatment settings including unknown diagnostic and treatment histories, limited efficacy and safety data about psychotropics, and finding existing algorithms and guidelines are not sufficient for this population. As youth enter PRTF on multiple medications and having not been successfully maintained in the community, we speculate that that there may be an underlying assumption that inter-class concurrent pharmacotherapy is to be expected. Physician training around medications has historically focused on initiation, titration and monitoring (levels, side effects) of medications, with less attention given to re-assessment of the risk/benefit or indications for discontinuation. In view of the unknown risk of long-term exposure to multi-class psychotropic medication, potential for drug-drug interactions and additive drug adverse reactions, coupled with the limited safety and efficacy data, implementing a deprescribing practice is warranted. Deprescribing guidelines would fill the gap identified in current guidelines.

The growth in antipsychotic medications is attributed to their use to address disruptive and aggressive behaviors, including such behaviors often present among those with ADHD. The diagnosis of ADHD does increase the likelihood of concomitant psychotropic use; however, it appears the ADHD diagnosis serves as proxy for maladaptive behaviors (e.g., aggression, behavioral dysregulation) frequently experienced by some youth with ADHD (27, 28). It is likely these associated behaviors that drive the use of concomitant psychotropic use. The T-MAY guidelines for maladaptive aggression in youth recommend that only after trials of psychosocial treatment and stimulants have been deemed ineffective should there be consideration for antipsychotics (29). In our study ADHD and DMDD were the two most prevalent diagnoses.

Stimulants are effective not only for ADHD but are also effective in controlling aggression. Our finding of greater concomitant use of alpha agonists and antipsychotics rather than alpha agonists and stimulants raises questions as to why optimizing treatment with stimulants was not preferred over antipsychotics, given their relative risks and benefits.

The only covariate found to be significantly associated with discharging on 4 or more medications was the number of medications present at admission. Connor et al.'s (16) study of the use of psychotropics in a residential treatment school found 70% of youth who had received trials of multiple concurrent pharmacotherapy prior to admission continued to receive such combinations at admission.

Some challenges exist in comparing our PRTF findings of concomitant psychotropic use to findings from other studies carried out in other residential settings given their heterogeneity noted earlier. Prior studies conducted in residential settings may have excluded youth with diagnoses of intellectual disability or psychotic disorders and utilized diagnostic criteria that predated DSM-5. The rates of concurrent psychotropics (3 or more) prescribed at time of admission from our study were comparable to earlier studies in residential settings. A number of studies that found a reduction in medication over time were explicit in their use of guidelines or included physicians with a shared approach to judicious prescribing (22, 24–28).

## Strengths

Our retrospective study utilized more recent Medicaid claims data, examined one level of care, PRTF, rather than a variety of residential settings that differ in the population served and mental health treatment services offered and provided data on inter-class polypharmacy and specific combinations of classes. Unlike previous residential studies of psychotropic utilization drawing on data from one or two facilities, our study encompassed 21 different PRTFs.

## Limitations

This study utilized Medicaid claims data of youth receiving PRTF services covered by CCBHO from the majority of counties in Pennsylvania and may not represent prescribing practices in residential treatment facilities in other regions. PRTFs in Pennsylvania are not evenly distributed across the state and can contract with multiple BHMCOs. We acknowledge the limitation however believe our data are representative of prescribing practices in PRTFs. We have no reason to believe that the prescribing psychiatrists in the PRTFs would alter their overall prescribing practices based on the BHMCO covering the youth. Administrative claims data do not provide detailed clinical information; symptom and behavioral data that may be associated with concurrent pharmacotherapy are not available. Claims data are valuable in providing estimates of prescribing patterns found in PRTFs, but it does not reveal why child and adolescent psychiatrists utilize concomitant psychotropic agents nor does it provide clinical outcomes data on the risks and benefits of combined medications for this population. We did not have information at the provider PRTF level regarding specific initiatives on reducing concomitant pharmacotherapy. Data about foster care and juvenile justice status for youth at the time of entry or exit from PRTF would have been useful; however, the state did not provide us with that information.

## Clinical, Research, And Policy Implications

Concomitant pharmacotherapy has become an accepted practice across levels of care and across age groups, though it is lacking strong evidence of its benefits with few exceptions and has associated risks. Our finding that significant proportions of youth in the most intensive level of care receiving 2, 3, and 4 or more classes of psychotropics provides data for future studies to evaluate the benefits and risks of common combinations. We endorse the call for additional research on complex inter-class regimens that has been made repeatedly (1, 4, 5, 7, 30). Research studies on the benefits and risk of combined treatment would address the evidence gap challenging psychiatrists providing clinical care, provide data regarding the effectiveness for populations, and tackle questions regarding the safety of chronic exposure to concurrent psychotropics.

Each day physicians must make clinical decisions with regards to concomitant pharmacotherapy; as such we advocate for the adoption of deprescribing practices by psychiatrists and other prescribing clinicians. Deprescribing is part of good prescribing practice, providing a systematic approach to identify and discontinue medications when the harms outweigh the benefits. The American Academy of Child and Adolescent Psychiatry (AACAP) practice parameter on the use of psychotropic medication includes two key principles relevant to deprescribing: (1) the need for a clear rationale for using medication combinations and (2) discontinuing medications requires a specific plan (31). Deprescribing has its origins in geriatric medicine (32) and since has gained attention by psychiatry (33) and child and adolescent psychiatry (34–38). The American Academy of Pediatrics (AAP) and AACAP appreciate that maltreated children are more likely to receive psychotropic medication than their peers and has issued guidance on trauma-informed assessment and pharmacologic treatment considerations (39). Gupta et al. (33) and Bellonci et al. (34) offer practical stepwise guidance on deprescribing.

State level quality improvement interventions initially focused on antipsychotic prescribing for children enrolled in Medicaid and/or in foster care and later expanded efforts to address polypharmacy. Strategies include antipsychotic prior authorization policies, mandatory peer review, and voluntary psychiatric consultation programs. As of 2018, 23 states and Washington DC offered telephonic psychiatric consultation, often aimed at primary care physicians (40). Evaluating the impact of these interventions is beyond the scope of this paper. One Medicaid statewide quality improvement program included both pediatricians and psychiatrists (in community mental health centers and residential treatment settings) and found a significant decrease in polypharmacy for the psychiatrist group (41). Managed care organizations may have opportunities to incentivize safe and judicious prescribing through the use of value-based performance contracting.

## Conclusion

Concomitant psychotropic pharmacotherapy is common practice in many PRTFs in Pennsylvania with antipsychotic, antidepressant, alpha-2-agonist, mood stabilizers, and stimulants frequently used in combination despite limited efficacy and safety data. These findings support adoption of deprescribing practices and support the call for publicly funded research on the effectiveness and safety of inter-class pharmacotherapy.

## Data Availability Statement

The original contributions presented in the study are included in the article/Supplementary Material, further inquiries can be directed to the corresponding author.

## Ethics Statement

The studies involving human participants were reviewed and approved by Study was approved by the UPMC (University of Pittsburgh Medical Center) Quality Review Committee. Written informed consent for participation was not required for this study in accordance with the national legislation and the institutional requirements.

## Author Contributions

GE contributed to the study design, writing, and literature review. KE contributed to the data analysis and revisions. MP contributed to the data analysis. NR contributed to study design and critiqued the manuscript, AH critiqued and edited the manuscript. All authors contributed to the article and approved the submitted version.

## Conflict of Interest

The authors declare that the research was conducted in the absence of any commercial or financial relationships that could be construed as a potential conflict of interest.
